# A double blind randomized trial of wound infiltration with ropivacaine after breast cancer surgery with axillary nodes dissection

**DOI:** 10.1186/1471-2253-11-23

**Published:** 2011-11-24

**Authors:** Axelle Vigneau, Anne Salengro, Joelle Berger, Roman Rouzier, Emmanuel Barranger, Emmanuel Marret, Francis Bonnet

**Affiliations:** 1 Department of Anaesthetics and Intensive Care, Tenon Hospital, Assistance Publique hôpitaux de Paris, University Pierre & Marie Curie, Paris VI. 4 rue de la Chine 75020 Paris, France; 2 Department of Gynaecologic Surgery and Obstetrics, Tenon Hospital, Assistance Publique hôpitaux de Paris, University Pierre & Marie Curie, Paris VI. 4 rue de la Chine 75020 Paris, France; 3 Department of Gynaecologic Surgery and Obstetrics, Assistance Publique Hôpitaux de Paris, Université Denis Diderot, Paris VII. 2, rue Ambroise Paré, 75010 Paris, France

## Abstract

**Background:**

The effect of local infiltration after breast surgery is controversial. This prospective double blind randomized study sought to document the analgesic effect of local anaesthetic infiltration after breast cancer surgery.

**Methods:**

Patients scheduled for mastectomy or tumorectomy and axillary nodes dissection had immediate postoperative infiltration of the surgical wound with 20 ml of ropivacaine 7.5 mg.ml^-1 ^or isotonic saline. Pain was assessed on a visual analogue scale at H2, H4, H6, H12, H24, H72, and at 2 month, at rest and on mobilization of the arm. Patient'comfort was evaluated with numerical 0-3 scales for fatigue, quality of sleep, state of mood, social function and activity.

**Results:**

Twenty-two and 24 patients were included in the ropivacaine and saline groups respectively. Postoperative pain was lower at rest and on mobilization at 2, 4 and 6 hour after surgery in the ropivacaine group. No other difference in pain intensity and patient 'comfort scoring was documented during the first 3 postoperative days. Patients did not differ at 2 month for pain and comfort scores.

**Conclusion:**

Single shot infiltration with ropivacaine transiently improves postoperative pain control after breast cancer surgery.

**Trial registration number:**

NCT01404377

## Background

Surgical wound infiltration with a local anaesthetic solution is currently performed in many surgical procedures including, abdominal hysterectomy, caesarean section and inguinal hernia repair [1]. Wound infiltration is reported to provide immediate postoperative pain control lasting for several hours [2]. In addition, long term benefits have been suggested such as prevention of chronic pain syndrome after surgery [1]. Breast cancer surgery is associated with mild to moderate pain but some procedures including axillary nodes dissection are more painful [3]. In these patients, pain may impair postoperative comfort and may prevent from mobilization of the corresponding upper limb. Moreover, studies have pointed out that chronic pain syndromes may develop after breast surgery that could be, at least partly, related to the intensity of acute postoperative pain and axillary nodes dissection [4]. Local infiltration with a local anaesthetic solution, has been studied on several instances with disappointing results, most of the studies documenting indeed the lack of significant effect of wound infiltration on postoperative pain after breast surgery [5]. Several flaws and methodological bias may question the results of these studies. For example, lidocaine, that is a very short acting anaesthetic agent, is used in most of the clinical trials [5]. In addition, in several trials breast infiltration is not combined with axillary infiltration [5]. Moreover, less painful procedures such as tumorectomy are mixed up with more painful ones. Petttersson et al. have indeed suggested that local infiltration is worth performing only in patients submitted to the most painful procedures [6]. Eventually, pain is measured at rest and not on movements which are considered more painful. These many reasons justify a well conducted prospective study aiming to assess the analgesic effect of a long lasting local anaesthetic used for complete infiltration of the surgical incisions.

## Methods

This prospective, double-blind, randomized, single centre study was approved by an institutional ethical committee (*Comité Consultatif de Protection des Personnes dans la Recherche Biomédicale Paris- Cochin, president: Pr. Guerin C, approval on October 11^th ^2005, registration number 2277*) and written informed consent of the patients was obtained. ASA I - II patients, older than 18 years, scheduled for unilateral mastectomy or tumorectomy associated with axillary nodes dissection were included in the study. Patients receiving opioid or any other analgesic treatment for chronic pain before surgery, patients with known allergy to local anaesthetics, and patients with acquired or genetic haemostatic abnormality were excluded.

On the morning of surgery, patients were allocated randomly into two groups, using a table of random numbers and already prepared sealed envelopes. In the treated group infiltration was performed with a ropivacaine 7.5 mg.ml-1 solution and in the control group with an isotonic saline solution. Solutions were prepared and provided by the anaeshetist in charge of the patient in the operating theatre, to the surgeon blinded for patient allocation. In the two groups patients were operated under general anaesthesia using propofol 2.5 mg.kg-1 and sufentanil 0.25 mcg.kg-1 for induction, and sevoflurane 1-1.5% and nitrous oxide 50% in oxygen for maintenance. Atracurium 0.6 mg.kg-1 was used for orotracheal intubation. Dexamethasone 4 mg was given intravenously after anaesthetic induction for prevention of postoperative nausea and vomiting. Twenty milliliters of the allocated solution were used, at the end of the surgical procedure, shared in two equal parts, to infiltrate the subcutaneous and deep layers of the breast and axilla surgical incisions. Infiltration was performed under direct vision by the surgeon. On awakening from anaesthesia, immediately at the end of surgery, patients were placed in a recovery room. Postoperatively, in case of pain, patients were instructed to use tablets of paracetamol 1g every 6 hours for 3 days. If pain was not controlled by paracetamol 5 mg of subcutaneous morphine were used as a rescue.

Pain intensity was measured on a visual analogue scale graded from 0 to 100. Measurements were recorded by nurses blinded for patient allocation, for pain at rest, and on maximum abduction of the operated arm (patients were asked to perform abduction of the arm as far as they could and pain intensity corresponding to this movement, was recorded). Pain measurements were performed at 2, 4, 6, 12, 24, 48, and 72 hour after the end of surgery. The value of maximum abduction angle was noted for each patient.

To evaluate quality of life patients were asked to score on a 4 points scale graded from 0 (the worst) to 3 (the best) the following items: sleep - fatigue - global activity - relationship with relatives - state of mood. A global score was attributed to each patient as the sum of categorical scores. Evaluation was performed at 24, 48 and 72 hour after the end of surgery.

Patients left the hospital between the fourth and the sixth postoperative day. They were evaluated at two month, during the postoperative surgical consultation, for residual pain at rest and on movement using a visual analogue scale and for quality of life as previously defined.

The sample size was based on an estimate of a 30% (SD 10%) decrease in pain intensity on mobilization on the day of surgery that was considered as the primary outcome. Given a type I error of 0.05 and a Type II error of 0.1, the required sample size was 22 patients per group; 25 patients were included in each group to take into account possible dropouts. Secondary outcomes were a decrease in pain score at rest, a decrease in rescue analgesic consumption and an improvement in the quality of life.

Statistical analyses were performed with Stata v10.0 software (Stata Corporation, College Station, TX, USA).". Results are expressed as mean+/- standard deviation (SD). Normally distributed data (VAS scores, abduction angles) were analyzed using a two-way analysis of variance. Categorical data (surgical procedure, operative side, adjuvant chemotherapy) were analyzed using Chi-2 test or Fisher's exact test. Mann-Whitney U-test was used for comparison of age, BMI, weight, and duration of surgery. P < 0.05 was considered significant.

## Results

**Figure 1:**
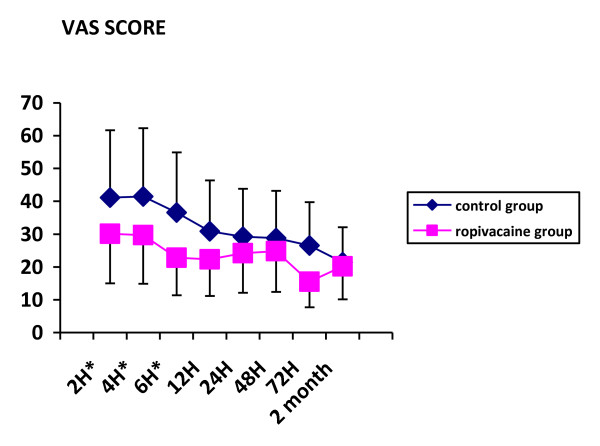
**VAS scores on mobilization of the arm on the operated side in the two groups of patients in the postoperative period and at two month**. * P < 0.05.

**Figure 2:**
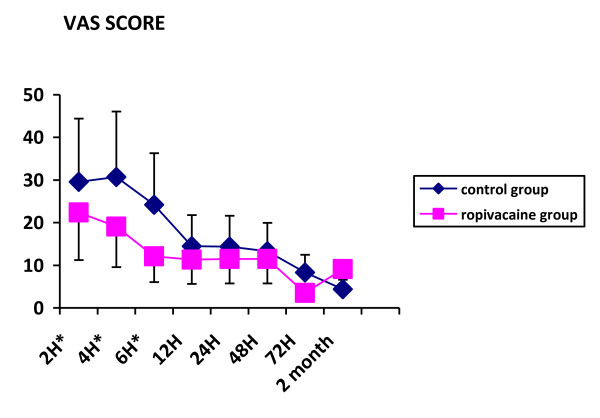
**VAS scores at rest in the two groups of patients in the postoperative period and at two month**. * p < 0.05.

**Figure 3:**
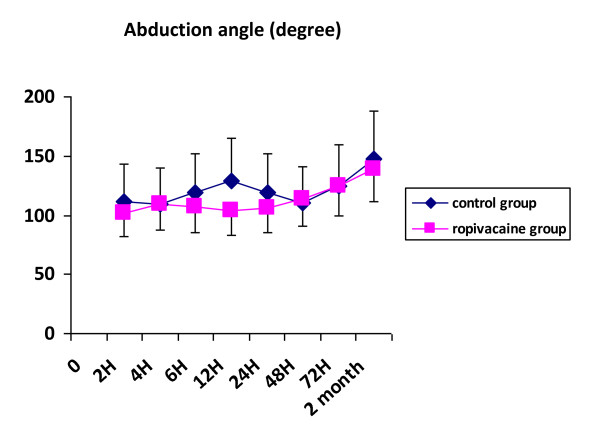
**Values of abduction angle of the arm in the postoperative period and at two month in the control and the ropivacaine groups**.

Three patients in the ropivacaine group and one in the control group were dropped out for surgical complication (one patient) and early discharge from hospital preventing from complete data collection (3 patients). Patients were comparable for demographics except for mean age which was statistically higher in the ropivacaine group (table 1). Types of surgical procedures and duration of surgery were comparable in the two groups. Measurement of pain on visual analogue scale documented lower scores at rest and on mobilization, in the ropivacaine group, 2, 4 and 6 hours after the end of the surgical procedure (Figures 1 &2). The difference was not significant afterward. Paracetamol consumption over the first three postoperative days was 5.7+/-3.4 g and 6.9+/-3.6 g in the ropivacaine and the control groups respectively. Two patients in the control group and 3 in the ropivacaine group received a 5 mg subcutaneous dose of morphine rescue medication. Values of arm abduction angle were comparable in the two groups at each time of measurement (Figure 3). Five patients in the ropivacaine group and 4 patients in the control group had nausea or vomiting. Mean score values for quality of sleep and for global assessment of quality of life were also comparable (Tables 2 &3). Five patients in each group complained of postoperative nausea.

**Table 1:** Demographics of the patients included in the data analysis; Values are expressed as mean +/- standard deviation.

	Control group N = 24	Ropivacaine group N = 22
**Age (yr)**	50 ± 11	58 ± 13*

**Weight (kg)**	67 ± 14	69 ± 11

**BMI**	25 ± 5	26 ± 5

**Operative side (R/L)**	13/11	10/12

**Duration of surgery (min)**	83 ± 25	126 ± 152

**Mastectomy/Tumorectomy + axillary nodes dissection**	17/7	17/5

**Adjuvant chemotherapy (Y/N)**	5/19	3/19

* = p < 0.05

**Table 2:** Quality of sleep after surgery.

	First 12H	First night	Second night	Third night
**Control group** **(N = 24)**	2.3 ± 0.8	2.3 ± 0.8	2.2 ± 0.8	2.6 ± 0.6

**Ropivacaine group** **(N = 22)**	1.9 ± 1.1	2.3 ± 0.8	2.6 ± 0.6	2.7 ± 0.6

Sleep is quoted on a 4 points scale from 0 (the worst) to 3 (the best imaginable). Values are expressed as mean +/- standard deviation

**Table 3:** Score of quality of life after surgery.

	12H	24H	48H	72H
**Control group** **(N = 24)**	10.3+2.4	10.7+2.4	10.6+1.9	12.0+1.5

**Ropivacaine group** **(N = 22)**	9.9+3.0	10.9+2.7	11.9+2.1	12.5+1.9

Postoperative fatigue, sleep, state of mood, relationship with relatives, and global activity are quoted on a 4 points scale from 0 (the worst) to 3 (the best imaginable). At each time of measurement the global score (maximum value: 15) is the addition of scores established for each parameter. Values are expressed as mean +/- standard deviation

Two months after surgery VAS scores at rest were 4.4+/-10.7 and 9.1+/-15.2 in the control and the ropivacaine groups respectively (NS). Values of VAS scores on mobilization were 21.4+/-4.1 and 20.2+/-4.0 in the control and the ropivacaine groups respectively. Arm abduction angles were 147+/-23° and 139+/-29° in the control and the ropivacaine groups respectively. At this time, the quality of sleep was comparable in the two groups (2.9+/-.3 and 2.7+/-.6 in the control and the ropivacaine groups respectively) and the groups did not differ for global evaluation of quality of life (13.0+/-.2 and 12.3+/-.4 in the control and the ropivacaine groups respectively)

## Discussion

This study documents significant but short duration benefit of wound infiltration after cancer breast surgery with axillary nodes clearance. Infiltration with ropivacaine solution was nevertheless effective for 6 hours after surgery but pain was controlled as well in patients without infiltration after this period of time. Since incomplete evaluation of pain and its consequences, were considered as flaws in previous studies, we carefully monitored pain, not only at rest but also on mobilization. We performed measurements of pain intensity soon after surgery, to evaluate the initial effect of infiltration. We assessed abduction of the upper limb knowing that it could be more painful after axillary incision, and we also evaluated patient's comfort. Nevertheless, local infiltration did not improve the ability to move the operated arm. Eventually, patient's comfort was equally effective when patients had been infiltrated or not.

The duration of the effect of ropivacaine infiltration was in fact comparable to that reported by Baudry et al [7]. and to the effect documented in other surgical procedures such as hernia repair [2]. Using a shorter acting local anaesthetic agent, i.e. lidocaine, Rosaeg et al documented a significant effect during less than 4 hours after non cancer breast surgery [8]. This has led others to use continuous wound infiltration after radical mastectomy to prolong the duration of pain control [9,10]. Nevertheless the use of an invasive analgesic technique is not always worth performing due to a rapid decrease in pain intensity as observed in the current study. We observed indeed that due to paracetamol administration, pain was comparably controlled in postoperative patients after 12 hours, even when they had not received ropivacaine infiltration. Paracetamol is a weak non opioid analgesic and the fact that pain was controlled by paracetamol administration supports the hypothesis that pain intensity was mild to moderate but not severe enough to the point of requiring additional analgesic technique during the first days after surgery. In other words, analgesia was easily achieved with paracetamol in most of the patients making the combination with continuous wound infiltration useless. In agreement only a few patients required rescue morphine in the two groups. A significant improvement in surgical technique over the past years, that makes it less invasive, traumatic and consequently less painful, may account for this matter of fact.

We looked at parameters that could be impaired by pain in the postoperative period. The incidence of nausea was comparable and quite low in the two groups thanks to the preoperative administration of dexamethasone. In addition, dexamethasone could also have contributed to postoperative pain control as previously documented [11]. Arm abduction angle that could be limited by pain in the axilla, was in fact quite high in both groups and increased comparably during the first postoperative days. We also assess quality of postoperative recovery through a composite index including fatigue, state of mood, activity, sleep, and relationship with relatives and found it also comparable in the two groups and only slightly altered. We especially report values of sleep's quality that was poorly disturbed in the postoperative period likely due, as for the cumulative score of quality of life, to adequate pain control.

The balance between advantages and drawbacks of local infiltration should consider the risk of adverse events. Although not reported in this series, the risk of inadvertent intravascular injection has to be kept in mind, prevented by careful aspiration before injection.

Although it has been claimed that infiltration and regional block may have long lasting effect and may prevent the occurrence of late pain after surgery especially after breast surgery [12], the results of this study do not support this hypothesis. In studies from Fassoulaki et al. regional anaesthetic technique was more complete, including thoracic nerve block and was also associated with adjuvant treatment [12,13]. Baudry et al. have looked for chronic pain after cancer breast surgery by telephone interview one year after surgery, and failed to document a decreased incidence in patients who had received local infiltration of the operative wound [7]. Thus, the direct preventive effect of local infiltration on late pain after surgery remains to be demonstrated.

Several other studies have also documented that regional anaesthesia using thoracic paravertebral block provided better pain control after unilateral breast surgery [14-17]. Most of the time surgical procedures were more important including breast reconstruction. Paravertebral block ensures a complete anaesthesia of the hemithoracic wall and the axilla that depends on T1 sensory distribution. Thoracic paravertebral block has been compared to local infiltration and demonstrated to be superior [9]. When indicated by a painful surgical procedure, thoracic paravertebral is therefore a better alternative than local wound infiltration for postoperative pain control.

One can argue that preoperative infiltration, before skin incision would had provide better results in terms of postoperative pain control. Nevertheless, preoperative wound infiltration has not been documented to be superior to postoperative infiltration in the specific setting of breast surgery [18]. This study has several limitations. One can argue that the number of patients was limited but it was calculated in agreement with the main outcome and allows robust conclusion concerning this outcome. Patient mean age was older in the ropivacaine group. Since increasing age may result in decreasing pain scoring and in decreasing the incidence of chronic pain after breast surgery, this difference, that occurred by random, may have contributed at least partly to the difference in pain scores [19]. The follow up was limited to two months but no difference in pain control was pointed out at this time making unlikely any further difference later on. No continuous infusion was used but VAS scores during the first postoperative days were adequately controlled as previously discussed.

## Conclusion

In patients scheduled for cancer breast surgery with axillary nodes dissection, wound infiltration with ropivacaine provides an effective pain control during a period of time limited to the first hours following surgery. No further benefit is documented then after.

## Competing interests

The authors declare that they have no competing interests.

## Authors' contributions

AV and FB designed the study-protocol; AV, AS, and JB included patients and checked for patients' files as investigators; RR and EB were referent surgeons and performed all the infiltrations; EM performed statitistics, FB wrote the manuscript, all authors reviewed and approved the manuscript.

## Pre-publication history

The pre-publication history for this paper can be accessed here:

http://www.biomedcentral.com/1471-2253/11/23/prepub
